# HDACi: The Columbus’ Egg in Improving Cancer Treatment and Reducing Neurotoxicity?

**DOI:** 10.3390/cancers14215251

**Published:** 2022-10-26

**Authors:** Angelica Squarzoni, Arianna Scuteri, Guido Cavaletti

**Affiliations:** 1 Experimental Neurology Unit and Milan Center for Neuroscience, School of Medicine and Surgery, Milano-Bicocca University, 20900 Monza, Italy; 2 PhD Program in Neuroscience, School of Medicine and Surgery, Milano-Bicocca University, 20900 Monza, Italy

**Keywords:** HDACs, HDAC inhibitors, HATs, HDAC6, HDAC5, nervous system, central nervous system, peripheral nervous system, neurons

## Abstract

**Simple Summary:**

The inhibitors of histone deacetylases (HDACs) enzymes are an emerging class of drugs proposed for the treatment of several tumors. Despite a general antineoplastic effectiveness, HDACs inhibitors showed conflicting results concerning their effects on the nervous system. Such a discrepancy could be ascribable to the different actions of the HDACs and to drug selectivity. In this review, we analyzed the role of the main HDACs within the nervous system to better understand the effect of their inhibition, with the aim of identifying the most promising candidate to become an effective antineoplastic drug with a limited neurotoxic profile.

**Abstract:**

Histone deacetylases (HDACs) are a group of enzymes that modify gene expression through the lysine acetylation of both histone and non-histone proteins, leading to a broad range of effects on various biological pathways. New insights on this topic broadened the knowledge on their biological activity and even more questions arose from those discoveries. The action of HDACs is versatile in biological pathways and, for this reason, inhibitors of HDACs (HDACis) have been proposed as a way to interfere with HDACs’ involvement in tumorigenesis. In 2006, the first HDACi was approved by FDA for the treatment of cutaneous T-cell lymphoma; however, more selective HDACis were recently approved. In this review, we will consider new information on HDACs’ expression and their regulation for the treatment of central and peripheral nervous system diseases.

## 1. Introduction

Epigenetics is the branch of genetics that studies the alterations in gene expression that are not caused by a change in DNA sequence, but rather result from environmental influences [1]. Such epigenetic modifications mainly act on histones, and they can be achieved in different manners: they can be derived from post-transcriptional modifications (PTMs) or be caused by microRNAs (miRNAs) and long non-coding RNAs (lncRNAs) [1,2,3]. Histones are a pivotal class of proteins in charge of both forming chromatin and changing its condensation state by a direct interaction with DNA. In fact, histones are positively charged proteins able to wrap the negatively charged DNA, thus forming the nucleosome; different nucleosomes are then linked together by histone 1 (H1) [1,4]. The structure of the histone’s nucleosome is formed by two dimers of histone 2A (H2A) and histone 2B (H2B) as well as one tetramer formed by histone 3 (H3) and histone 4 (H4), around which 146 base pairs (bp) of DNA are wrapped. As key proteins for chromatin structure and function, histones serve as regulators of gene expression and every change in chromatin state may have a critical role in gene expression [4]. Although not directly involving a change in DNA sequence, epigenetic modifications can be inherited by progeny, and overall, they can be responsible for various pathologies [1].

As stated before, histone activity may be regulated in different manners and in particular by PTMs, which can be divided into four main categories: phosphorylation, acetylation, methylation and ubiquitination [5,6]. In this review, we will focus on acetylation and, in particular, on deacetylation.

## 2. HDACs and HATs

**Figure 1:**
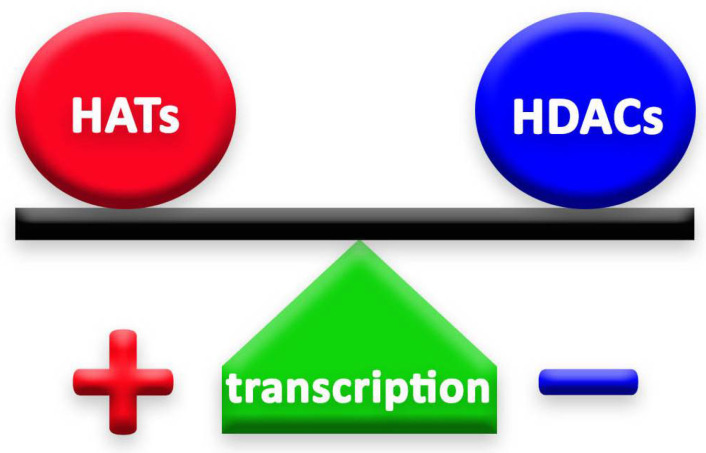
The balance between HATs and HDACs is crucial to the well-being of cells and organisms. The action of HDACs lowers gene transcription. On the contrary, when HDACs are repressed, the effect of HATs is stronger and leads to higher levels of gene transcription.

Histone deacetylases (HDACs) and histone acetyltransferases (HATs) are two groups of enzymes that play opposite roles in epigenetic mechanisms. Through their deacetylation and acetylation processes, respectively, these enzymes have a counteractive role on lysine, thus affecting chromatin organization [4] (Figure 1). Histone deacetylation, induced by HDACs, enhances the ionic interaction between histones and DNA, thus consequently leading to a tighter chromatin state [7]. In this way, HDACs secure chromatin and, therefore, limit gene accessibility to transcription factors, leading to lower transcription levels [8]. HDACs exist in multiprotein complexes and interact with different DNA-binding elements [7].

HDACs are also referred to as “erasers”, as their action leads to a decreased gene expression [3]. Enzymes that accomplish this function can be divided into four different categories (class I, class II, class III and class IV), based primarily on their homology to histone deacetylases expressed in *Saccharomyces cerevisiae*. For example, Class II HDACs have homologous structures to the HDA1 proteins of yeast [9].

This classification can be further branched based on the substance that is required for their action: zinc-dependent HDACs (class I, II and IV) or NAD+-dependent HDACs (class III, also known as Sirtuins) [10]. HDACs can be expressed in the nucleus, in the cytoplasm or they can shuttle in both parts. In some cases, just the transport of these enzymes from nucleus to cytoplasm leads to cytotoxicity in the cell.

On the contrary, histone acetyltransferases (HATs) transfer acetyl groups from acetyl coenzyme A (Acetyl Co-A) to the ε-amino group of lysine residues, thus promoting a less condensed state of chromatin and therefore enhancing gene transcription. Their action is not restricted to histone proteins [11].

The balance of HDACs’ and HATs’ activity in the cell is fundamental for the achievement of the correct cellular function [12] and, for this reason, these enzymes need to be strictly regulated in the cells (Figure 1). Any mutation or any change in their expression, or in the expression of the genes they regulate, could even lead to tumor development and cause neurological disorders, such as Huntington’s (HD) and Alzheimer’s diseases (AD) [13,14].

Originally, HDACs and HATs were given these names because the scientific community believed that these enzymes could only act on histones. Currently, due to their action on non-histone proteins as well, HDACs are also named lysine deacetylases (KDACs) and HATs are named lysine acetyl transferases (KATs) [1]. In this review, we are going to address them as HDACs and HATs.

## 3. HDAC Inhibitors (HDACis)

Due to their involvement in tumor development, different authors have explored HDACs as targets for antineoplastic treatments, thus leading to the discovery that HDAC repression could be exploited for the treatment of a number of pathologies [1,2,3,4], not only malignancies, but also other diseases with a known acetylation impairment, such as neurodegenerative diseases [1,5,6].

In 2006, FDA authorized the first HDACi for cancer treatment, namely SAHA (Vorinostat) [7]. After SAHA, three other HDACis were approved by the FDA (see Table 1). All these drugs can be considered pan-HDACis, as they do not inhibit an HDAC’s class specifically [1]. Theoretically, all these drugs can act on all HDACs, leading to the disrupting or protective events in cells and tissues. For this reason, even if their action is supported by clinical and research data, the diffuse actions of pan-inhibitors can lead to various side effects and this is one of the reasons for the interest of researchers on the shift from pan to selective HDACis [8].

Currently, more specific HDACis have been designed, and are currently under study both in pre-clinic and clinical models. So far, several studies indicate that the HDACi specificity enhances drug activity and accuracy in the treatment. The use of HDACi, and in particular of HDAC6 inhibitors, has been suggested for the treatment of different neurological diseases [5,9,10,11,12]. The putative therapeutic potential of HDAC6 inhibitors has been reported in different experimental models, both in vitro and in vivo; in these models, the use of slightly different HDAC6i has made it possible to obtain encouraging results, with the recovery of cognitive abilities in models of Alzheimer’s disease [10] and fragile X syndrome [11]. Moreover, by acting on tubulin dynamics and restoring axonal transport, these inhibitors counteract motor dysfunctions in Charcot–Marie–Tooth disease [12], in Rett syndrome [13] and in a model of amyotrophic lateral sclerosis [5].

Therefore, the possible use of the existing HDACis in the field of nervous system diseases treatment is intriguing, although still controversial. This point is further strengthened by a simple observation: some of the new compounds that inhibit HDACs, and are currently exploited for their antineoplastic effects, also showed a reduced neurotoxicity profile, or even neuroprotective properties [14]. By modulating the activity of some HDACs, it could be theoretically possible to both exploit their already ascertained antineoplastic properties, and to explore their neuroprotective potential. This could be a turning point for both the oncological and neurological fields, since the use of some antineoplastic drugs is often limited by their neurotoxic side effects [15]. The identification of an anti-tumor agent without a neurotoxic activity or even with neuroprotective properties, to be used alone or in combination, could dramatically impact anticancer treatment. So far, several papers investigated the antineoplastic potential of HDACis [16,17,18,19], and in this review we want to spotlight their activity on the nervous system. To better understand the potential of such molecules for the treatment of neurological diseases (including those of toxic origin, such as chemotherapy-induced peripheral neurotoxicity), we present an overview of the different HDACs and their putative role in neuronal and glial cells.

### 3.1. Localization of Different HDACs in Neurons

**Figure 2:**
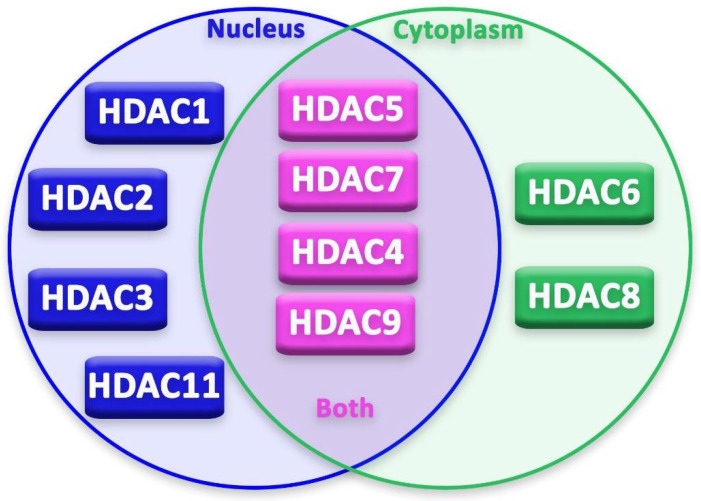
Localization of HDACs in the cell.

Epigenetic regulation in neurons and glia cells is strictly regulated by several epigenetic modifiers and transcription factors [20]. Moreover, durable changes, both in mature and “immature” neurons, occur through post-translational alterations in histones [20]. In recent years, advancement in basic research on HDACs’ expression, localization (Figure 2) and activity in neurons has been achieved by studies on neurodegenerative diseases. These studies focused on the possible neuroprotective or neurotoxic effect of different HDACs on neurons and other cells of the nervous system.

**Figure 3:**
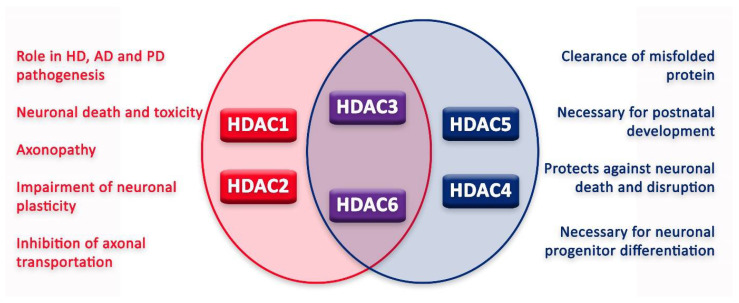
Graphic illustration of roles of HDACs in neurons.

In the nervous system, HDACs can have various functions, sometimes leading to contrasting outcomes (Figure 3). Moreover, for some HDACs, the literature still fails to reconcile a unanimous effect on the nervous system and its cells. However, since the approval of HDACis, new tests have been conducted in order to elucidate HDAC function and HDACi application in the treatment of different nervous system diseases.

### 3.2. HDAC1 and HDAC2: Neurotoxicity at Its Finest

HDAC1 and HDAC2 are two enzymes expressed in mammalian cells within the nervous system, part of class I and homologous of Rpd3 expressed by *Saccharomyces cerevisiae* [8,21].

HDAC1 is highly expressed in the cerebellum, amygdala and hippocampus. Recent studies suggested that HDAC1 is expressed in neurons, astrocytes and oligodendrocytes [22,23,24], even if with contrasting effects: some authors suggested that, in neurons, it acts as an inhibitor of axonal transport, since it is able to bind motor proteins, causing mitochondrial trafficking impairment [22], thus suggesting for HDAC1 a neurotoxic effect. In particular, HDAC1 export from nucleus to cytoplasm seems to be the main cause of mitochondrial movement dysfunctions in neurons. The transportation to cytoplasm depends on the interaction between HDAC1 and nuclear Exportin 1 receptor (CRM1) and the cytosolic expression of HDAC1 is reported in damaged axons of multiple sclerosis patients [25], indicating that its export to the nucleus is necessary for its neurotoxic effect.

A recent study on Parkinson’s disease (PD) indicates that the abnormal transportation of HDAC1 from the nucleus to the cytoplasm could be one of the causes of the axonopathy and abnormal motor behavior in PD patients [26]. This observation has been confirmed by research on HD, since its neurotoxicity is linked to its transportation to the cytoplasm in an HD model [9] and in a multiple sclerosis model [25].

However, other papers point to HDAC1’s neuroprotective effect, such as toward hippocampal stem cell differentiation [27,28,29], and even more when HDAC1 functions in cooperation with other enzymes and proteins. The interaction between HDAC1 and a truncated form of HDAC9, a histone deacetylase-related protein (HDRP), seems to protect against neuronal death. HDAC1 is recruited by HDPR, leading to c-Jun deacetylation, and this deacetylation blocks neuronal death and apoptosis [28]. Another study confirmed that HDPR is usually downregulated in the process of neuronal death and high levels of HDRP inhibit the HDAC1 and HDAC3 interaction, preventing neurotoxicity [29].

Concerning the peripheral nervous system (PNS), a study on neuropathic pain observed that HDAC1 inhibition through opioids has been able to limit neuropathic pain in patients [30].

In the PNS, HDAC1 and HDAC2 are thought to be necessary for the myelination and survival of Schwann cells through the regulation of SRY-box transcription factor 10 (Sox10), pivotal for neural stem cells growth and migration, and early growth response 2 (Krox20) expression, with a central role in Schwann cell myelination [24,31].

Similarly to HDAC1, HDAC2 is highly expressed in the hippocampus and cerebellum [32], and exclusively in the nucleus. On one hand, it seems to have a role in neuronal progenitors’ differentiation during adult neurogenesis [27]. On the other hand, other studies reported it as a negative regulator of memory formation and synaptic plasticity [33,34].

A recent study suggested that HDAC2 is involved in AD and, through its inhibition, the synaptic plasticity may be re-established in the brain of the patients [35], hinting that an HDACi specific for HDAC2 could be a treatment for AD.

### 3.3. HDAC3: Neuron’s Grim Reaper

HDAC3 is part of HDAC’s class I, again homologous of Rpd3 expressed by *Saccharomyces cerevisiae* [8,21].

It is mainly expressed in the brain, both in neurons and in glial cells [32], with an important role in neurogenesis and gliogenesis [36,37,38].

Indeed, in vivo studies in HDAC3 knockout mice have resulted in death within 24 h and abnormalities in neuron localization [39]. A more recent study on the topic demonstrates the involvement of HDAC3 in the determination of oligodendrocyte vs. astrocyte differentiation [40]. More in detail, Zhang et al. identified HDAC3 as the epigenetic regulator for differentiation and maintaining of the oligodendrocyte phenotype [39,40]. More findings on the topic suggest that the commitment in differentiation derives from a coordinate action of HDAC3 and p300, one of the HATs. HDAC3 represses the promoter of genes that are required for astrocyte commitment and p300 represses the ones that are required for oligodendrocyte commitment [40].

Despite the role of HDAC3 during differentiation, of oligodendrocytes in particular, a large part of the literature suggests a neurotoxic role of HDAC3 in adult neurons. More in detail, it could act as a nucleus–cytoplasm shuttle [36], and that can operate as a neurodegeneration promoter by inducing inflammation and neurodegenerative processes [22]. HDAC3 overexpression is linked to higher rates of cortex and cerebellum neuronal death, apoptosis and toxicity [41]. Moreover, recent studies indicate that HDAC3 expression promotes neurodegenerative diseases such as PD, AD and HD. In the case of HD and AD, further in vitro and in vivo studies confirmed these data, underling that HDAC3′s inhibition protected from cognitive decline [17,42].

Such a neurotoxic effect has been confirmed by several studies using HDAC3 inhibition. HDAC3 knockout mice in vivo models and the use of a selective inhibitor of HDAC3, named RGFP136, have demonstrated to enhance long-term memory in a persistent manner [43]. In another in vivo model of HD, the involvement of HDAC3 is linked to aberrant transcriptional patterns, expansion in the huntingtin (Htt) gene and the negative regulation of genes involved in cognitive functions [19]. HDAC3 inhibition seemed protective towards both motor symptoms of HD and striatal volume changes in mice [18]. In this case, the selective inhibitor of HDAC3 showed a protective effect toward both glial cell and astrocyte activation in the mice model when compared to the wild-type mice [18]. Moreover, it was effective against long-term memory impairment and against the accumulation of mutant Htt, leading to a normalized expression of genes related to memory in the hippocampus [19].

To summarize, HDAC3 can have a strong neurotoxic action on adult neurons in the brain, leading to neurodegenerative diseases, such as HD. For these reasons, it is currently an attractive target to inhibit for possible future studies on neurodegenerative diseases [41], such as for AD treatment. Nevertheless, it could be of great interest to discern its epigenetic regulator role in neurogenesis and gliogenesis.

### 3.4. HDAC4: The Indecisive

HDAC4 is part of HDACs, class IIa. It is mainly expressed in the cytoplasm and its shuttle action is signal-dependent [22]. The shuttle between the nucleus and the cytoplasm of HDAC4 seems to be regulated by potassium and glutamate concentrations outside the neuron. HDAC4 is found in the cytoplasm of neurons but it shuttles to the nucleus, leading to the repression of survival factors for neurons, such as the myocyte enhancer factor 2 (MEF2) and the cAMP response element-binding protein [44].

In the brain, it is expressed in cells of the cerebellum, hippocampus and olfactory bulb [32]. Currently, its role is not clear and the scientific literature suggests that HDAC4 can have both neurotoxic and neuroprotective effects, without endorsing one of the two sides specifically.

Mielcarek and coll. observed an association between HDAC4 and mutant Htt in HD, with the formation of cytoplasmic inclusions [45]. HDAC4 inhibition seems to be protective toward the formation of such aggregates, synaptic functions in the hippocampus and symptoms of HD in a mouse model [45]. However, the same authors also reported that nuclear aggregates were still present with the inhibition of HDAC4 [45], suggesting that it may not be the only pathway involved in the formation of aggregates.

HDAC4 could also exert a neuroprotective activity: in a mouse model lacking HDAC4, CDK1 was highly active and caused structural abnormalities of Purkinje neurons postnatally. Moreover, recent studies have shown that it is essential for the postnatal development of the brain, although this action seems to not be related to the deacetylation [46,47].

### 3.5. HDAC5: Neuron’s Healer

HDAC5 is an enzyme, part of class IIa, that shuttles between the cytoplasm and the nucleus of cells of both the CNS and PNS [32]. All data suggest a neuroprotective effect for this enzyme, carried out by the inhibition of apoptosis [48] and the enhancement of axonal regeneration and growth after injury [27,48]. This neuroprotective effect derives from its affinity for microtubules and its ability to reduce microtubules’ acetylation [49]. In particular, HDAC5 is able to promote microtubules’ regeneration, after an axonal injury by tubulin deacetylation [49]. Tubulin deacetylation is caused by calcium influx and requires HDAC5 in order to be executed [49]. HDAC5 exits the nucleus, goes into the neuron’s cytoplasm and has an anterograde movement into the axon [48]. These findings suggested that only through HDAC5 is nuclear export axonal regeneration permitted, while it does not occur if HDAC5 is still trapped inside the nucleus [48]. This study suggested that HDAC5 may act as a switch for axonal regeneration in the PNS, but not in CNS injuries [48]. A possible explanation for this difference resides in the finding that there is no shuttle of HDAC5 from the nucleus to the cytoplasm in the CNS cells, probably leading to a failure during the regeneration [48]. Despite this, the research on HDAC5 focuses more on the mechanisms leading to a protective effect against injuries in the PNS. A study on AD indicates that the inhibition or loss of HDAC5 relates to the impairment of memory functions, likely inhibiting the possible role of HDAC5 in memory consolidation [50].

To conclude, these findings caused a rise in research on this specific HDACi, leading to a new hope in the treatment of microtubule dynamics in dorsal root ganglia (DRG) injuries, while HDAC5 expression and function in CNS neurons still remains an open question.

### 3.6. HDAC6: The One to Rule Them All?

HDAC6, part of class IIb, is composed of a C-terminal ubiquitin-binding domain and an N-terminal composed of two homologous catalytic sites [22,51,52]. Besides histones, it also has different substrates, such as acetylated tubulin in polymerized microtubules and heat shock protein 90 (Hsp90) [53].

HDAC6 is still puzzling researchers for its different roles in different pathways, possibly being both neuroprotective and neurotoxic at the same time, depending on the state of the cell. Its role is considered neurotoxic due to its modulation of microtubule-dependent cell movement and its action on α-tubulin, consequentially affecting the transport of neurotrophic factors and other proteins in the neuron [25,54,55]. The HDAC6 neurotoxic hypothesis states that this enzyme removes ubiquitinated proteins from the axon and may also deacetylate α-tubulin as a side effect, promoting axonal transport defects and participating in neurodegeneration [56]. Due to these data supporting the HDAC6 neurotoxic effect, several studies have investigated the effects of its inhibition: some authors demonstrated that the inhibition of HDAC6 led to an increase in microtubule acetylation [57]. This action could be exploited as a possible treatment for HD, wherein the microtubules are hypoacetylated and microtubule transport is impaired. In addition, regarding microtubule transport impairment, it has been reported that SAHA increased the vesicular transport of the brain-derived neurotrophic factor (BDNF) by inhibiting HDAC6’s action [58].

HDAC6 inhibition is currently under study as a treatment for chemotherapy-induced peripheral neuropathies (CIPNs). CIPNs affect patients treated with classical chemotherapy drugs and can affect the patient in both the short- and long-term, affecting the PNS. One of the classical chemotherapy drugs is vincristine, a vinca alkaloid that acts on microtubules, leading to the inhibition of cancer cells proliferation [59]. Since HDAC6 can act towards α-tubulin, specific inhibitors were tested in mice and rat models in addition to vincristine. A humanized mouse model for T-cell acute lymphoblastic leukemia was applied to verify that the use of the selective inhibitor for HDAC6 reduces the antineoplastic effect of vincristine. The combination of these two drugs on these rodent models led to a significant benefit in the behavioral tests and did not reduce the antineoplastic effect [59]. Such a combination, effective for both the antineoplastic and protective effect towards CIPNs, is considered particularly encouraging [59].

Other studies are focusing on the damaging action that HDAC6 exerts towards mitochondria, being a central component of autophagy and controlling the autophagosome–lysosome fusion [60]. For this reason, it is one of the most studied HDACs, in the hope to find a specific treatment for AD and PD [61,62], whose pathogenesis is partially caused by mitochondrial impairment. This new notion led to more studies to identify a selective HDACi towards HDAC6. In vivo, in a mouse model for AD with lower HDAC6 expression, required learning and memory and α-tubulin acetylation, the inhibition of HDAC6 seems to ameliorate cognitive decline [63]. Moreover, recent in vivo studies in mice models suggested that the decrease in HDAC6 expression led to a re-establishment of memory and learning functions in mice that expressed low levels of alpha-tubulin acetylation [1].

It is worth noting that, despite the seeming neurotoxic effect, some studies suggest that HDAC6 may be implicated in axonal regrowth due to its effect on alpha-tubulin, which it deacetylates [52,56], and some authors reported that HDAC6 plays a role against the accumulation of misfolded and aggregated proteins [64,65]. HDAC6 in the cytoplasm seems to coordinate the clearance of protein aggregates and autophagic degradation and, moreover, it regulates the cell protective response to cytotoxic protein aggregate formation, probably by dissociating the HDAC6/HSF1/HSP90 complex, activating HSF1 and the major cellular chaperones [66]. If HDAC6 is deleted, it is possible to observe neurodegeneration and the impossibility of disposing of protein aggregates [60]. Moreover, HDAC6 can also deacetylase the protein tau, which results in acetylated in neurofibrillary tangles [57].

To conclude, HDAC6 plays a main role in the regulation of tubulin acetylation and in mitochondrial impairment. A few papers indicate a possible role in the clearing of misfolded proteins, possibly being neuroprotective.

Moreover, a study identified HDAC6-specific inhibitors as a possible treatment for CIPNs [59].

### 3.7. Other HDACs

Besides the above-mentioned HDACs, there are very limited data on the role of HDAC7, HDAC8, HDAC9, HDAC10 and HDAC11 in nervous cells. For some of them, such as HDAC8, a role in neuronal differentiation has been suggested [64], while HDAC9 and HDAC11 activity has been related to neuronal survival, neurite elongation and axonal development [27,67]. The opposite effect has been suggested for HDAC10, which has been reported to block neuronal differentiation [68], while the expression of HDAC7 in astrocytes promoted inflammation through Nf-kB activation [69]. So far, however, only a few studies on these isozymes have been conducted, and new insights on the role of such HDACs would be necessary to understand their possible implication in the nervous system.

## 4. Conclusions

In general, HDACis are a promising treatment for different kinds of tumors; however, more studies on their possible application for the diseases of the nervous system are necessary. Currently, some clinical trials targeting HDACis for both neurodegenerative diseases and psychiatric disorders are ongoing (ClinicalTrials.gov Identifier: #NCT02124083) [1], and a few new trials have been implemented in recent years in order to test the already existing and approved HDACis for the treatment of neurodegenerative and psychiatric diseases. Some of them, such as SAHA, can also pass the blood–brain barrier (BBB) [1], opening new opportunities to treat neurodegenerative disorders by modifying gene expression directly in CNS neurons and glia. Here, we offered an overview on the role of the different HDACs in the nervous system, in order to stress the neuroprotective potential of some of them, which could be exploited not only for neurodegenerative diseases, but also to counteract CIPNs. In this way, we suggested the possible link between the antineoplastic and neuroprotective actions of HDACis: a treatment that helps the patient overcome the disease while being protected against the neurotoxic side effects.

As illustrated in this review, current publications on the topic are conflicting regarding the possible protective or detrimental role of HDACs in the nervous system. This indicates that a better understanding of the basic mechanisms underlying HDACs’ function in neuronal cells and glial cells is necessary, in order to elucidate their possible application in the clinical practice.

As reported before, in the current literature HDAC5 appears to be the only HDAC to have an indisputable neuroprotective action, and specific inhibitors against HDAC5 could be counterproductive due to its positive role in axon restoration after injuries. In addition to HDAC5, a few published papers indicate that HDAC4 also has a neuroprotective effect.

On the other hand, the suppression and inhibition of HDAC1-2-3’s action could be promising since researchers suggested that these three enzymes have a definite neurotoxic effect in the cells of the nervous system. Regarding HDAC1 and HDAC2, only a few papers support their neuroprotective action towards neuronal death and neurogenesis, respectively, and it is important to remark that HDAC3 still has an unclear role in the differentiation of oligodendrocytes, which may be worthy of further studies.

Indeed, HDAC6 seems to play a controversial role, bringing together both a possible neuroprotective and neurotoxic effect. Seemingly, current research suggests that its effects on the CNS can be neuroprotective, while the ones on the PNS are neurotoxic. To date, there is no explanation for this apparent different action, which is likely related to a differential expression of HDAC6 in these two systems.

The ambiguity of HDAC6’s role in the topic of neuroprotection persists, but a few papers, such as the one by Van Helleputte et al., seem to indicate that the inhibition of HDAC6 is neuroprotective in the PNS [59]. Moreover, the work of Van Den Bosch et al. shows that the expression of HDAC6 may lead to neurodegeneration caused by an impairment in axonal transportation [56].

For these reasons, it is possible to deduce that the main role of HDAC6 is a neurotoxic one and its inhibition may protect against neurotoxicity.

A final consideration should be made on the clinical use of HDACis: a current limit for the clinical application of HDACis for neurological diseases surely resides in their general side effects, but also on the limited knowledge of the molecular basis of HDACis’ effect on nervous cells [70]. Moreover, it should be considered that some of these HDACis possess a genotoxic potential, derived by the presence of hydroxamic acid in their zinc-binding group [71,72]. Although such a risk could be accepted during cancer treatment, a more careful use must be considered for the long-term treatments necessary for neurodegenerative diseases. Some authors have already faced this point and bypassed the problem by selecting more selective inhibitors, with a reduced genotoxic profile and a better ability to cross the BBB [12].

In conclusion, the deep knowledge of the effects of HDACis paves the way to their application for the treatment of different diseases affecting both the CNS and the PNS. In order to achieve this goal, it is important to focus the current research on the basic mechanisms underlying HDACs’ action, thus contributing to the design of more specific HDACis, as those specifically targeting HDAC6, to better exploit all their effects, both antineoplastic activity and neuroprotection.

## Figures and Tables

**Table 1:** List of FDA-approved HDAC inhibitors, with year of approval, treatment and administration.

HDACi Name	Year of Approval (FDA)	Approved for (FDA)	Primary HDAC Targeted	Administration
SAHA (Vorinostat) (Zolinza)	2006	Cutaneous T-cell lymphoma (CTCL)	HDACs class I and HDAC6	Oral
Panobinostat (Farydak)	2015	Refractory MM	pan-HDACi	Oral
Belinostat (Beleodaq)	2014	T-cell lymphoma	pan-HDACi	Intravenous
Romidepsin (Istodax)	2009	Cutaneous T-cell lymphoma	HDAC6	Intravenous
